# Structural Characterization and Repair Mechanism of *Gracilaria lemaneiformis* Sulfated Polysaccharides of Different Molecular Weights on Damaged Renal Epithelial Cells

**DOI:** 10.1155/2018/7410389

**Published:** 2018-08-05

**Authors:** Da Guo, Kai Yu, Xin-Yuan Sun, Jian-Ming Ouyang

**Affiliations:** Institute of Biomineralization and Lithiasis Research, Jinan University, Guangzhou 510632, China

## Abstract

Natural *Gracilaria lemaneiformis* sulfated polysaccharide (GLP0, molecular weight = 622 kDa) was degraded by H_2_O_2_ to obtain seven degraded fragments, namely, GLP1, GLP2, GLP3, GLP4, GLP5, GLP6, and GLP7, with molecular weights of 106, 49.6, 10.5, 6.14, 5.06, 3.71, and 2.42 kDa, respectively. FT-IR and NMR results indicated that H_2_O_2_ degradation does not change the structure of GLP polysaccharides, whereas the content of the characteristic −OSO_3_H group (13.46% ± 0.10%) slightly increased than that of the natural polysaccharide (13.07%) after degradation. The repair effects of the polysaccharide fractions on oxalate-induced damaged human kidney proximal tubular epithelial cells (HK-2) were compared. When 60 *μ*g/mL of each polysaccharide was used to repair the damaged HK-2 cells, cell viability increased and the cell morphology was restored, as determined by HE staining. The amount of lactate dehydrogenase released decreased from 16.64% in the injured group to 7.55%–13.87% in the repair groups. The SOD activity increased, and the amount of MDA released decreased. Moreover, the mitochondrial membrane potential evidently increased. All polysaccharide fractions inhibited S phase arrest through the decreased percentage of cells in the S phase and the increased percentage of cells in the G2/M phase. These results reveal that all GLP fractions exhibited repair effect on oxalate-induced damaged HK-2 cells. The repair ability is closely correlated with the molecular weight of the fractions. GLP2 with molecular weight of about 49.6 kDa exhibited the strongest repair effect, and GLP with higher or lower molecular weight than 49.6 kDa showed decreased repair ability. Our results can provide references for inhibiting the formation of kidney stones and developing original anti-stone polysaccharide drugs.

## 1. Introduction

Seaweed polysaccharides possess an extensive range of biological activities [1]. However, native seaweed polysaccharides cannot easily penetrate the cell membrane to exert their biological activity because of their large molecular size and poor solubility [2, 3]; as such, these polysaccharides have limited applications. The biological activity of polysaccharides with high molecular weight may be improved by degradation. Jo and Choi [4] performed degradation of *Sargassum fulvellum* polysaccharide to obtain three polysaccharide fractions with low molecular weights of 2, 23, and 36 kDa; the antioxidant and anticoagulant activities increased with decreasing molecular weight of the polysaccharides. Zhu et al. [5] revealed that sulfated fucoidan with low molecular weight (5–7 kDa) showed higher anticoagulant effect than that of sulfated fucoidan with molecular weight of 120–82 kDa.

*Gracilaria lemaneiformis*, which belongs to Rhodophyta, Florideae, Gigarfinales, Gracilariaceae, and Gracilaria, is widely distributed in the south coastal areas of China and coastal areas near Japan and Korea [6] *G. lemaneiformis* is consumed as food in many Asian countries and mainly used in food industries as gelling agent [7]. *G. lemaneiformis* polysaccharide (GLP) mainly consists of alternating 3-linked *β*-D-galactopyranosyl agarose with −OSO_3_H and 4-linked *α*-L-galactopyranosyl carrageenan units [6]. The 3-linked unit belongs to the D-series, and the 4-linked unit may have the D or L configuration, often occurring as a 3,6-anhydrogalactopyranosyl moiety [6–8]. Many reports are available with regard to the chemical structure and biological activity of GLPs. GLP exerts many beneficial bioactivities, such as antitumor, antiviral, and antioxidant activities and hypoglycemic properties [6, 9, 10]. Fan et al. [9] isolated an acidic polysaccharide (GLSP) with carbohydrate content of 72.06% and sulfate content of 6.13% from *G. lemaneiformis*; the GLSP significantly inhibited the growth of tumor, promoted splenocyte proliferation and macrophage phagocytosis, and increased the levels of IL-2 and CD8^+^ T cells in blood of tumor-bearing mice. The results suggest that the isolated GLSP displayed remarkable antitumor and immunomodulatory activities. Liao et al. [6] studied the hypoglycemic and antioxidant effects of a polysaccharide extracted from *G. lemaneiformis* (GLP; Mw, 121.89 kDa). The intragastric administration of GLP for 21 d induced an obvious decrease in the blood glucose level. Furthermore, GLP evidently increased the activities of superoxide dismutase and glutathione peroxidase and total antioxidant capacity and significantly decreased the level of malondialdehyde in the liver, pancreas, and kidney of diabetic mice. Di et al. [10] extracted a crude polysaccharide of *G. lemaneiformis* (GRPS) by hot water extraction and obtained three purified polysaccharides, namely, GRPS-1-1, GRPS-2-1, and GRPS-3-2, with average molecular weights of 1310, 691, and 923 kD, respectively. All the polysaccharides exhibited antioxidant effects, including clearance of ABTS and superoxide radicals and inhibition of lipid peroxidation.

The incidence of kidney stone has gradually increased in recent years [11, 12]. Currently, the main prescription drugs for treatment of urinary calculi are citrate, magnesium preparations, orthophosphate, allopurinol, and thiazide diuretics. However, the action mechanism of these drugs remains unclear, and their curative effects can be marginal [13]. Thus, scholars must develop new highly efficient, nontoxic, and inexpensive anti-stone drugs for scientific and practical applications [14].

Oxalic acid is a metabolism product of the human body and a main component for the formation of kidney stones. When oxalic acid in urine reaches a certain concentration, human kidney proximal tubular epithelial cells (HK-2 cells) will be oxidatively damaged [15], which is correlated with the formation of kidney stones [16, 17]. The damaged cells can be repaired by plant polysaccharides [18, 19].

In our previous study [18], we have studied the effect of sulfate group (−OSO_3_H) content of six kinds of seaweed polysaccharides (SPSs) on repair ability to damaged HK-2 cells. The six SPSs were extracted from *Laminaria japonica*, *Porphyra yezoensis*, *Gracilaria lemaneiformis* (GLP), *Sargassum fusiforme*, *Eucheuma gelatinae*, and *Undaria pinnatifida*, respectively. The six SPSs have a narrow difference of molecular weight (from 3343 to 4020 Da), and their sulfate group (−OSO_3_H) content was 21.7%, 17.9%, 13.3%, 8.2%, 7.0%, and 5.5%, respectively. The results showed that the repair ability of polysaccharide was positively correlated with −OSO_3_H content. Polysaccharide with higher −OSO_3_H content exhibited a better repair ability on damaged cells. In addition, the molecular weight is also an important factor affecting the viability of polysaccharides. However, the antioxidant activity and repair effect of GLP with different molecular weights on HK-2 cells have not been investigated yet. In the present study, natural GLP was degraded by H_2_O_2_ to obtain eight GLP fractions with different molecular weights ranging from 2.42 kDa to 622 kDa. The repair effect of the fractions on oxalate-induced damaged HK-2 cells was also studied to elucidate the mechanism of kidney stone formation and provide experimental evidence for development of new anti-stone drugs.

## 2. Experiments

### 2.1. Reagents and Instruments

*Gracilaria lemaneiformis* sulfated polysaccharide (GLP0) was produced by Beijing New Probe Bioscience & Technology Co., Ltd (Beijing, China). Samples of *G. lemaneiformis* were collected from the Qingdao province of China from September to December 2016. The material was sorted, washed, and dried immediately by forced air circulation at 50–60°C.

The cell proliferation assay kit Cell Counting Kit-8 (CCK-8) and lactate dehydrogenase (LDH) assay kit were purchased from Dojindo Laboratories (Kumamoto, Japan). Hematoxylin and eosin (HE) staining kit, 5,5′,6,6′-tetrachloro-1,1′,3,3′-tetraethylbenzimidazolylcarbocyanine iodide (JC-1) kit, and propidium iodide (PI) were purchased from Shanghai Beyotime Bioscience & Technology Co., Ltd. (Shanghai, China). Hydrogen peroxide, KBr (SP), and other chemical reagents were of analytical grade and purchased from Guangzhou Chemical Reagent Company (Guangzhou, China) and D_2_O from Sigma (99.9%). Experimental water is secondary distilled water.

The apparatus used include an enzyme mark instrument (SafireZ, Tecan, Switzerland), upright fluorescence microscope (22DI-E-D282, Leica, Germany), flow cytometer (FACSAria, BD company, USA), FT-IR spectrometer (Equinox 55, Bruker, Germany), ultraviolet-visible spectrophotometer (Cary 500, Varian company, USA), conductivity meter (DDS-11A, Leici, Shanghai, China), and NMR spectrometer (Varian Bruker 300 MHz, Germany).

### 2.2. Preparation of *G. lemaneiformis* Polysaccharides

Algal powder of *G. lemaneiformis* (diameter, 200 *μ*m) was subjected to hot water extraction with 90-fold volumes of distilled water for 5 h at 90°C according to the method described by Liao et al. [6] to obtain polysaccharide. After centrifugation to remove residues (7000 rpm, 10 min), the supernatant was concentrated to one-third of its volume in a vacuum rotary evaporator. The concentrated solution was precipitated with three volumes of absolute ethanol overnight at 4°C. The precipitates were collected by centrifugation (3500*g*, 10 min) and resolved in warm water. Proteins were removed using the Sevag method. The supernatant containing the polysaccharide was dialyzed in distilled water for 72 h and vacuum freeze-dried.

### 2.3. Degradation of Polysaccharides

Natural GLP0 was degraded using previously reported methods [20]. Briefly, 1.2 g of crude polysaccharide (GLP0) was weighed accurately and dissolved in distilled water at 70°C. After heating to 90°C, the reaction system was quickly added with 0.1%, 0.4%, 1%, 3%, 6%, 10%, and 15% H_2_O_2_ solution. The degradation reaction was allowed to proceed for 2 h, at which point the solution pH was adjusted to 7.0 by adding 2 mol/L NaOH solution. The degraded solution was concentrated to one-third of its original volume at 60°C. The product was precipitated by adding anhydrous ethanol three times. The solution was stored overnight and filtered. The filtrate was dried in vacuum to obtain the degraded polysaccharide. Productivity can be calculated after weighing the polysaccharide.

### 2.4. Measurement of Average Molecular Weights (*M*_r_) of Polysaccharide

According to the reference [21], the viscosity of a sample in an aqueous solution was measured using an Ubbelohde viscosity method at 30 ± 0.2°C. After measuring the fall time of polysaccharide before and after degradation in the viscometer, specific (*η*_sp_) and relative (*η*_r_) viscosity was calculated according to the formulas *η*_r_ = *T*_i_/*T*_0_ and *η*_sp_ = *η*_r_ − 1, where *T*_0_ and *T*_i_ are the falling time of deionized water and GLP solutions, respectively. *η* = (2(*η*_sp_ − ln*η*_r_))^1/2^/*c*, where *c* is the sample concentration. The *M*_r_ of GLP was calculated through its *η* value. *η* = *κM*_r_^*α*^, where *κ* and *α* are constants. For GLP, *κ* = 0.07 and *α* = 0.72 [21].

### 2.5. Analysis of Sulfate Group Content

The sulfate group (−OSO_3_H) content of GLP was measured by the BaCl_2_-gelatin turbidity method [18, 22]. The polysaccharide sample of 70 mg was placed in 10.0 mL of 1.0 mol/L HCl solution, then hydrolysated for 6 h at 100°C. After cooling, the HCl solution was added to the calibration line. A 0.3% gelatin solution is prepared in hot water (60 ~ 70°C) and stored at 4°C overnight. 2 g of BaCl_2_ was dissolved in a gelatin solution and left at room temperature for 2–3 hours. 0.2 mL of GLP solution with the concentration of 1.4 mg/mL was added to 1 mL of BaCl_2_-gelatin reagent and 3.8 mL of 0.5 mol/L HCl. After that, the mixture was allowed to stand at 25°C for 10–20 minutes. The blank was prepared by substituting 0.2 mL of water for the GLP solution. The released BaSO_4_ suspension was measured at *λ* = 360 nm by a UV-VIS spectrophotometer using K_2_SO_4_ as standard, and the regression equation is *Y* = 0.01042 + 1.27905*X*, *n* = 11, and *R* = 0.99324, from which the percentage of sulfate content of polysaccharide can be calculated.

### 2.6. Analysis of Carboxyl Content

The carboxyl (−COOH) content of GLP is determined by conductometric titration [18, 23]. The conductivity titration curve was plotted using the conductivity value as the *y*-axis and the corresponding volume of consumed NaOH as the *x*-axis. The conductivity titration curve can be divided into three parts, namely, the conductivity decrease phase (A), the balance phase (B), and the conductivity increase phase (C). Three tangents were constructed from the three-stage curves, and the intersections were stoichiometric points. The intersection of the A line and the B line gives the volume of NaOH (*V*_1_) such that excess HCl and −OSO_3_H are consumed, and the intersection of line B and line C gives the volume of NaOH (*V*_2_), excess HCl, and co-consumed GLP's −OSO_3_H and −COOH; therefore, *V*_2_ − *V*_1_ (platform portion) is the volume of consumed NaOH by −COOH alone of GLP. The −COOH content can be obtained according to the following formula:

(1) $$\begin{array}{c} -COOH\left(\%\right)=\frac{C_{NaOH}\times \left(V_{2}-V_{1}\right)\times 45/1000}{C_{sample}\times 40/1000}\times 100. \end{array}$$

*C*_NaOH_ (mol/L) represents the molar concentration of NaOH, *C*_sample_ (g/L) represents the mass concentration of GLP polysaccharide, 45 g/mol is the molar mass of −COOH, and 40 mL is the volumetric solution of polysaccharide. The final value is the average of three parallel experiments.

### 2.7. Fourier-Transform Infrared Spectroscopy (FT-IR) Analysis of GLP [24]

FT-IR spectra of polysaccharides were determined using films prepared by the dried polysaccharides and KBr pellets on a Fourier-transform infrared spectrophotometer in the wave number range of 4000–400 cm^−1^ with a resolution of 4 cm^−1^.

### 2.8. ^1^H and ^13^C NMR Spectrum Detection [24]

Approximately 20 mg of purified GLP was dissolved in 0.5 mL of deuterium oxide in an NMR tube (5 mm diameter), and analysis was performed using a Varian Bruker 300 MHz spectrophotometer. The chemical shifts recorded were given in parts per million (ppm).

### 2.9. Cell Viability Assay of Polysaccharide on HK-2 Cells

The HK-2 cells were cultured in DMEM medium containing 10% fetal bovine serum at 37°C and 5% CO_2_ humidified environment. When 80% to 90% confluent monolayers were reached, cells were lightly blown after trypsinization to form cell suspensions for the following cell experiments.

One hundred microliters of cell suspension with a cell concentration of 1 × 10^5^ cells/mL was inoculated per well in 96-well plates and incubated in a 5% CO_2_-humidified atmosphere at 37°C for 24 h. The culture medium was removed by suction, and cells were divided into four groups as follows: (A) cell-free culture medium group (control group of background); (B) control cells without polysaccharide treatment (sample control group); (C) damaged group of oxalic acid: the serum-free medium containing 2.8 mmol/L oxalic acid was added and incubated for 3 h; and (D) repair group: serum-free medium containing 60 *μ*g/mL GLP with different molecular weight was added to damaged cells and repaired for 10 h. Each experiment was repeated in three parallel wells. Then, the medium was changed to fresh serum-free DMEM culture medium and 10 *μ*L CCK-8 was added to each well and incubated for 1.5 h at 37°C. Absorbance (A) was measured by using the enzyme mark instrument at 450 nm. Cell viability was calculated based on the following equation.

(2) $$\begin{array}{c} Cell\;viability\left(\%\right)=\frac{A\left(treatment\;group\right)}{A\left(control\;group\right)}\times 100\%. \end{array}$$

### 2.10. Lactate Dehydrogenase (LDH) Release Assay

One hundred microliters of cell suspension with a cell concentration of 1 × 10^5^ cells/mL was inoculated per well in 96-well plates and incubated for 24 h. Then, the cells were divided into five groups in which the first 4 groups were divided as in Section 2.9, and the E group (E) was added: (E) cells without GLP treatment for the subsequent cleavage of the wells (sample maximum enzyme activity control wells). After repair for 12 h, the absorbances were analyzed at 490 nm according to the LDH kit instruction. LDH release (%) was calculated using the formula as follows:

(3) $$\begin{array}{c} LDH\%=\frac{A\left(group\;D\right)-A\left(group\;A\right)}{A\left(group\;C\right)-A\left(group\;A\right)}\times 100\%. \end{array}$$

### 2.11. Hematoxylin and Eosin (He) Staining

One milliliter of cell suspension with a cell concentration of 1.5 × 10^5^ cells/mL was inoculated per well in 12-well plates and incubated for 24 h. The cells were divided into three groups according to Section 2.9: (A) control group, (B) damaged group of oxalic acid, and (C) repair group of GLP. After the repair effect was completed, the supernatant was removed by suction and the cells were washed twice with PBS. The cells on the plate were fixed with 4% paraformaldehyde for 15 min and stained by hematoxylin and eosin according to the manufacturer's instruction. The cellular morphological changes were observed under microscope, and the nuclei were stained in violet and cytoplasm in pink or red.

### 2.12. Superoxide Dismutase (SOD) Activity and Malondialdehyde (MDA) Content Detection

The cell suspension with a density of 1 × 10^5^ cells/mL was plated per well in 96-well plates and incubated in DMEM containing 10% fetal bovine for 24 h. The cells were divided into three groups: (A) control group, (B) damaged group of oxalic acid, and (C) repair group of GLPs. Cellular SOD activity and MDA content were determined by using the SOD and MDA kits, respectively, according to the instructions provided with kits.

### 2.13. Measurement of Mitochondrial Membrane Potential (Δ*ψ*m)

One milliliter of cell suspension with a concentration of 4.0 × 10^5^ cells/mL was inoculated per well in 6-well plates for 24 h. After the cells were synchronized, the cells were grouped as in Section 2.9. Incubated for 12 h, the supernatant was aspirated and the cells were washed twice with PBS and digested with 0.25% trypsin. The cells were suspended by pipetting, followed by centrifugation (1000 rpm, 5 min). The supernatant was aspirated, and the cells were washed with PBS and centrifuged again to obtain a cell pellet. The cells were resuspended by adding and thoroughly mixing 500 *μ*L of PBS in a microcentrifuge tube. Finally, the samples were stained with JC-1 and then analyzed.

### 2.14. Cell Cycle Progression Assay

Cell concentration and group were the same as in Section 2.9. The medium was changed to serum-free DMEM culture media and then incubated for another 12 h to achieve synchronization. The cells were blown and suspended, followed by centrifugation (1000 rpm, 15 min). The supernatant was aspirated, and the cells were washed with PBS and centrifuged again to obtain a cell pellet. Afterwards, 300 *μ*L PBS containing 10% serum was added to resuspend cells and precooling 700 *μ*L absolute alcohol was added with constant shaking, sealed with sealing film, and stored at 4°C overnight. Then, cells were centrifuged at 2000 rpm for 5 min, and supernatant was aspirated. 500 *μ*L PBS was added to wash cells, followed by centrifugation again, and supernatant was removed. Finally, 200 *μ*L PI was added and mixed at 37°C for 15 min. The cell cycle was analyzed by a flow cytometer.

### 2.15. Statistical Analysis

Experimental data were expressed as mean ± SD. The experimental results were analyzed statistically using SPSS 13.0 software. The differences of means between the experimental groups and the control group were analyzed by Tukey's test. *p* < 0.05 indicates significant difference; *p* < 0.01 indicates extremely significant.

## 3. Results

### 3.1. Degradation of *G. lemaneiformis* Polysaccharide

**Figure 1:**
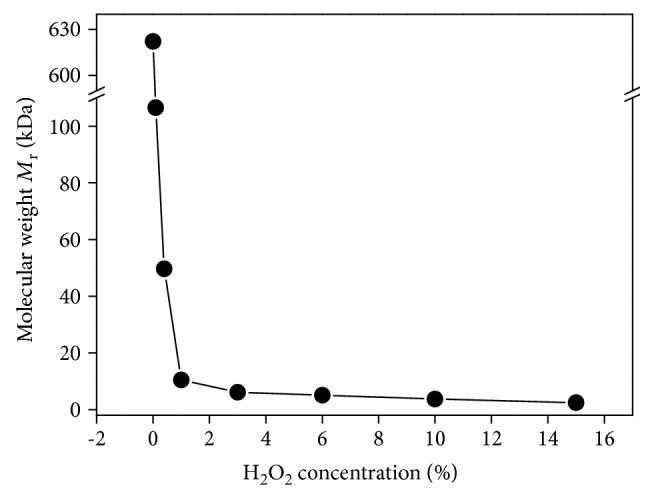
Relationship between H_2_O_2_ concentration and molecular weight of degraded GLPs.

Seven degraded polysaccharide fractions were obtained from crude *G. lemaneiformis* polysaccharide (GLP0) by using different concentrations of H_2_O_2_ (Table 1). When the concentration of H_2_O_2_ (*c*(H_2_O_2_)) was 0.1%, the molecular weight of the degraded products decreased from 622 kDa (GLP0) to 106 kDa, indicating that GLP0 was easily degraded. The molecular weight of the polysaccharide quickly decreased to 49.6 and 10.5 kDa when the H_2_O_2_ concentration added was increased to 0.4% and 1%, respectively (Figure 1). The molecular weight slowly decreased to 6.14 and 2.42 kDa when the H_2_O_2_ concentration was increased to 3% and 15%, respectively.

### 3.2. Change in the Contents of −OSO_3_H and −COOH Groups of the Polysaccharides before and after Degradation

**Figure 2:**
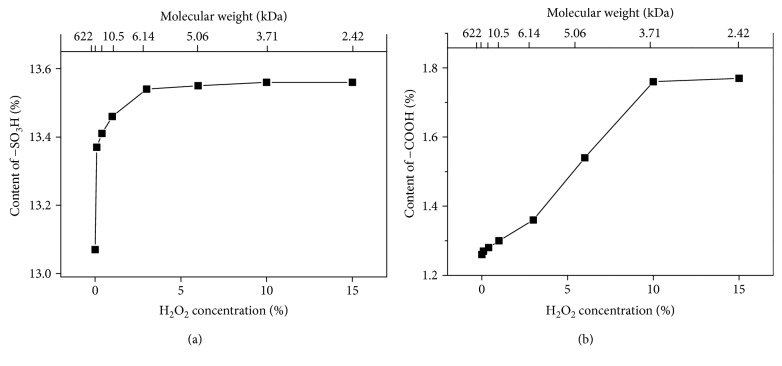
Relationship between the contents of −OSO_3_H and −COOH groups and the molecular weight of GLPs. (a) –OSO_3_H group and (b) –COOH group.

The **−**OSO_3_H content of each degraded polysaccharide fraction was about 13.46% (Figure 2(a)), which is slightly higher than 13.07% of natural GLP (Figure 2(a)). Similarly, the **−**COOH content of the degraded fractions was 1.27%–1.77%, which is higher than 1.26% of the crude GLP. Moreover, the **−**COOH content increased with decreasing molecular weight of the polysaccharides (Figure 2(b)), but the increase was not obvious.

### 3.3. Structural Characterization of GLP by FT-IR Spectra

**Figure 3:**
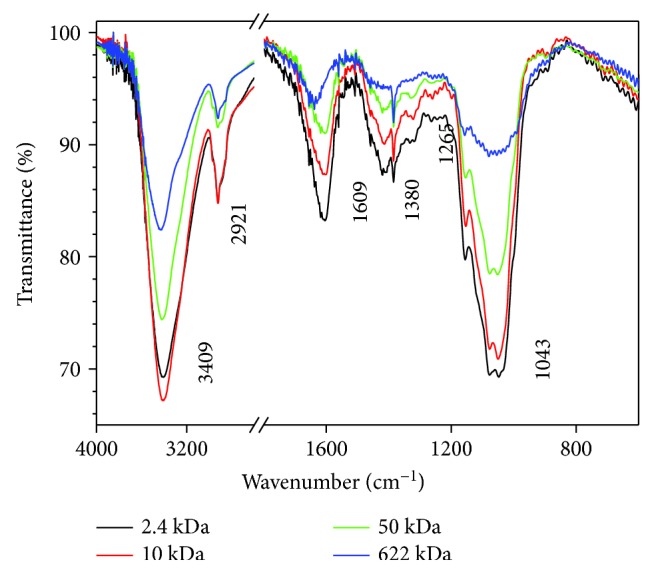
FT-IR spectra of different molecular weights of GLP.

The FT-IR spectra of polysaccharide from *G. lemaneiformis* before and after degradation were similar (Figure 3, Table 2), the peak intensity of GLP after degradation was increased, and no new peaks appeared, which indicated that the degradation of H_2_O_2_ did not cause a significant effect on the overall structure of polysaccharides.

The peak of 3409–3432 cm^−1^ was caused by the stretching vibration of O-H, and 2920–2928 cm^−1^ was caused by the stretching vibration of C-H; the peak near 1380 cm^−1^ was caused by the deforming vibration of C-H bond. The peak at about 1625 cm^−1^ was attributed to the asymmetric and symmetrical stretching vibration of −COOH [24, 25]. The peak at 1370 cm^−1^ was the signal area of ester sulfate [26], and the peak near 1260 cm^−1^ corresponds to S=O vibration of the sulfate groups [27]. The absorption near 1053 cm^−1^ and 1043 cm^−1^ was due to the stretching vibration of C-O, while the absorbance at 930 cm^−1^ is weak, indicating that galactose is substantially free of internal ether type [26].

### 3.4. ^1^H and ^13^C NMR Spectrum Analysis

**Figure 4:**
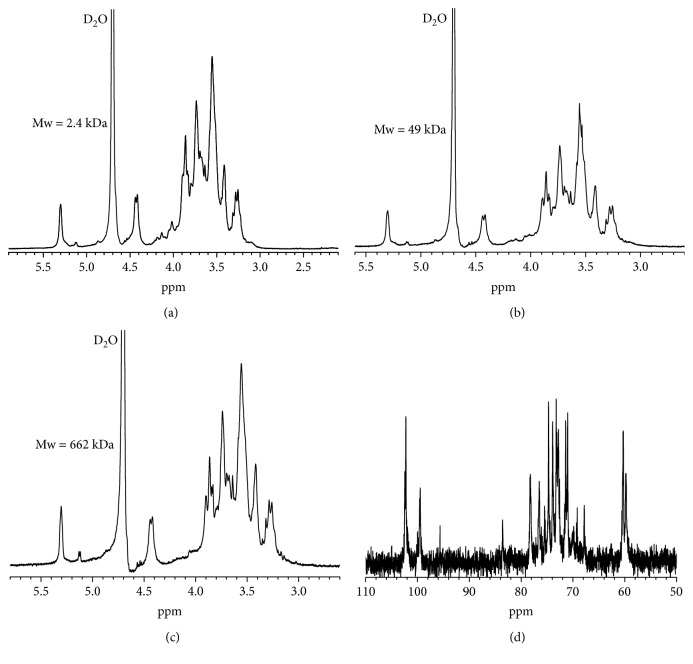
NMR spectra of GLP. (a–c) ^1^H NMR spectra and (d) ^13^C NMR spectrum.

Figure 4 shows the NMR spectra of four GLP. The NMR spectra of polysaccharides before and after degradation were similar, which indicated that the degradation of H_2_O_2_ did not cause a significant effect on the overall structure of GLP. The signal from the anomeric proton at *δ* 4.43 was assigned to H-1 of *β*-D-galactose, and the signal at *δ* 5.34 was attributed to anomeric proton of 3,6-*α*-L-galactose. The peaks at 930 cm^−1^ of FT-IR spectra are weak, indicating that the GLP contains little endogenous ether galactose [26]. It indicated that GLP consists of *β*-D-galactose and 6-O-sulfate-3,6-*α*-L-galactose, which is different with earlier reported literatures [6–8]. According to the number and the chemical shift value of ^13^C NMR in the 95–110 interval, the number of sugars and the conformation of the glycosidic bond in the oligosaccharide and its glycoside can be deduced [26]. As shown in Figure 4(d), there are two main peaks in the heterogeneous carbon region *δ* (95–110 ppm): the terminal carbon C-1 of *β*-D-galactose is at *δ* 102.35 ppm, and the terminal carbon C-1 of 3,6-*α*-L-galactose is at *δ* 99.48 ppm (Table 3).

### 3.5. Changes in Viability of HK-2 Cells after Repair by GLP with Different Molecular Weights

**Figure 5:**
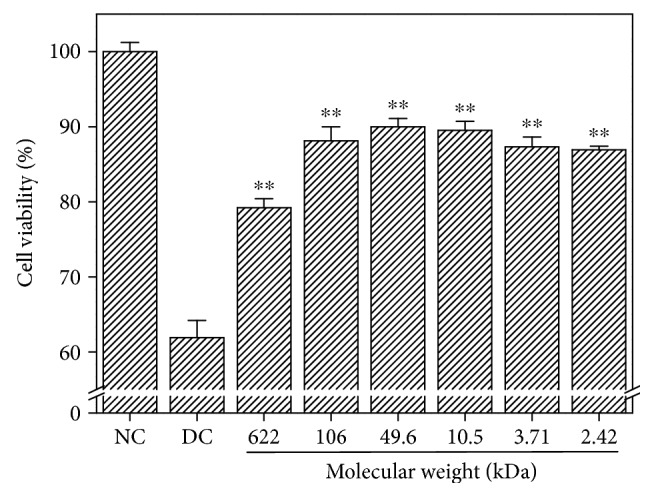
Cell viability of damaged HK-2 cells after being exposed to six GLP fractions with molecular weights of 622, 106, 49.6, 10.5, 3.71, and 2.42 kDa. NC: normal control; DC: damaged control by 2.8 mmol/L oxalate. GLP concentration: 60 *μ*g/mL. Compared to DC group: ^∗∗^*p* < 0.01.

Six GLP fractions with molecular weights of 622, 106, 49.6, 10.5, 3.71, and 2.42 kDa were used to repair oxalate-induced damaged HK-2 cells. In our preliminary study [18], we found that when damaged cells were repaired by different concentrations (20, 40, 60, 80, and 100 *μ*g/mL) of polysaccharides, the cell viability of damaged cells was initially increased, reaching the maximum at 60 *μ*g/mL and then decreasing at higher concentrations (100 *μ*g/mL), indicating that 60 *μ*g/mL was adequate for the polysaccharides to play a role. Thus, 60 *μ*g/mL of GLP was used to repair damaged HK-2 cells. The changes in cell viability after repair are shown in Figure 5. The cell viability of the damaged cells was only 61.9%, which increased to 79.2%–89.5% after treatment with various polysaccharides. The repair ability of nondegraded polysaccharide (GLP0) with molecular weight of 622 kDa was the weakest, and cell viability after repair was 79.2%. The repair ability of the degraded fraction GLP2 with molecular weight of 49.6 kDa was the strongest, and the cell viability after GLP2 repair was 89.5%. When the molecular weight of GLP was higher or lower than 49.6 kDa, the repair ability was reduced. The larger the deviation of the molecular weight from 49.6 kDa, the weaker the repair capacity of the polysaccharide will be.

### 3.6. Changes in Lactate Dehydrogenase (LDH) Release after Repair by GLP with Different Molecular Weights

LDH release is an important indicator of the integrity of the cell membrane. LDH is located in the cytoplasm under normal conditions [28]. When cells are attacked by foreign materials, the structure of the cell membrane will be destructed and cytoplasmic enzymes, such as LDH, will be released into the cell culture media. Thus, cytotoxicity can be quantitatively analyzed by detecting the amount of LDH released into culture media to evaluate the integrity of the cell membrane [28].

**Figure 6:**
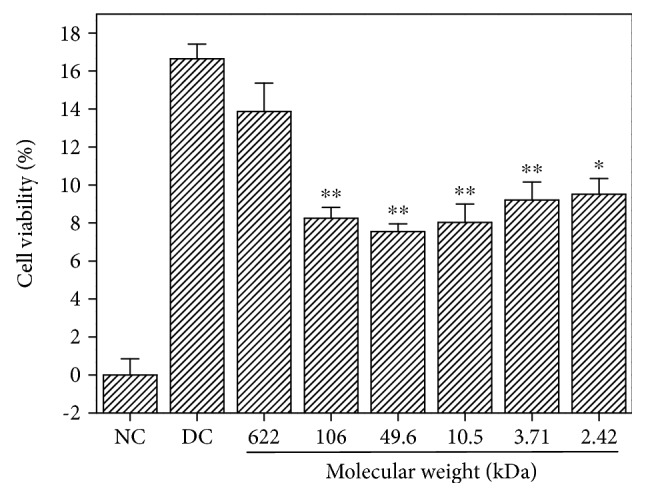
Changes in the amount of LDH released from the damaged HK-2 cells after treatment with six GLP fractions with different molecular weights. NC: normal control; DC: damaged control by 2.8 mmol/L oxalate. GLP concentration: 60 *μ*g/mL. Repaired time: 12 h. Compared to DC group: ^∗^*p* < 0.05 and ^∗∗^*p* < 0.01.

Figure 6 shows the changes in the amount of LDH released from the damaged HK-2 cells after treatment with six GLP fractions with different molecular weights. Compared with the damaged group (16.64%), the amount of LDH released decreased at different degrees (7.55–13.87%) after being repaired with various polysaccharide fractions. This finding indicates that GLP with different molecular weights exhibited a repair effect on the membrane of damaged HK-2 cells. Moreover, the amount of LDH released was the lowest (7.55%) after the cells were repaired by GLP2. Hence, GLP2 exhibited the strongest repair effect. When the molecular weight of various polysaccharide fractions was higher or lower than 49.6 kDa, the repair effect of the polysaccharides decreased. These results are in accordance with the findings on cell viability detected by the CCK-8 kit (Figure 5).

### 3.7. Cell Morphology Observation by Hematoxylin-Eosin Staining

Hematoxylin, an alkaline dye with a positive charge, stains the chromatin of the nucleus and the ribosome of the cytoplasm in violet. Eosin, an acidic dye with a negative charge, stains positively charged proteins in the cytoplasm and extracellular matrix in pink or red.

**Figure 7:**
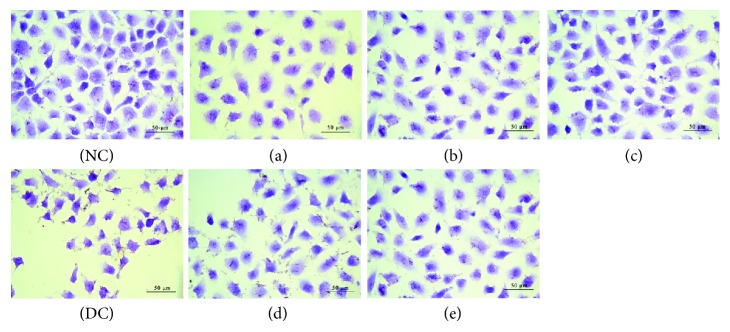
Cell morphology observation of damaged HK-2 cells by hematoxylin-eosin staining after treatment with six GLP fractions with different molecular weights. NC: normal control; DC: damaged control by 2.8 mmol/L oxalate; (a) GLP0, (b) GLP1, (c) GLP2, (d) GLP3, and (e) GLP6. GLP concentration: 60 *μ*g/mL. Repaired time: 12 h.

As shown in Figure 7, junctions between normal HK-2 cells were tight and the cells were plump. When HK-2 cells were exposed to oxalate (2.8 mmol/L) for 3 h, the cells lost their natural shape, the cell number evidently decreased, and the cells become concentrated. After being repaired by GLP with different molecular weights, the number of cells with intact shape increased, and the number of damaged condensed cells decreased (Figures 7(a)–7(e)). After the damaged cells were repaired by GLP2, the cell number reached the maximum and the morphology of the repaired cells became closest to the normal cells (Figure 7(c)). The repair effect of polysaccharides with molecular weights higher or lower than 49.6 kDa was weaker than that of GLP2.

### 3.8. Effect of GLP Repair on Superoxide Dismutase (SOD) Activity

**Figure 8:**
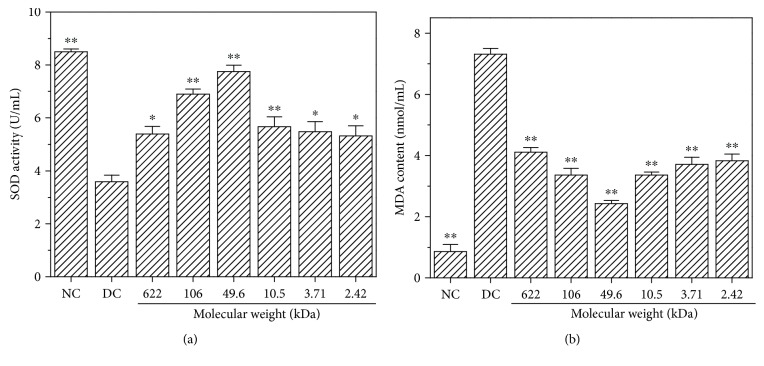
SOD activity (a) and MDA content (b) of the damaged HK-2 cells after treatment with six GLP fractions with different molecular weights. NC: normal control; DC: damaged control by 2.8 mmol/L oxalate. GLP concentration: 60 *μ*g/mL. Repaired time: 12 h. Compared to DC group: ^∗^*p* < 0.05 and ^∗∗^*p* < 0.01.

SOD activity can reflect the function of the antioxidant system. After HK-2 cells were injured by oxalate, the SOD activity decreased to 3.59 ± 0.25 U/mL, suggesting that the antioxidant capacity of the cells decreased (Figure 8(a)). The extracellular SOD of the repair group was all higher than that of the injury group, indicating that GLPs can repair cells by resisting oxidative damage. GLP2 with a molecular weight of about 49.6 kDa showed the strongest antioxidant capacity on injured cells.

### 3.9. Effect of GLP Repair on Malondialdehyde (MDA) Generation Amount

Changes in MDA content can reflect the degree of lipid peroxidation in the biomembrane.  Figure 8(b) shows the generation amount of MDA in the control, injured, and repaired group cells. The released MDA of injured cells increased, indicating that oxalic acid produces cell oxidative damage. The MDA amount in the repair groups was lower than that of the injury group, indicating that the oxidative damage level of cells decreased. In particular, GLP2 with a molecular weight of about 49.6 kDa showed the strongest repair effect; the amount of MDA was significantly (*p* < 0.01) reduced to 2.43 ± 0.10 nmol/L compared with that of the injury group (7.31 ± 0.19 nmol/L).

### 3.10. Changes in Mitochondrial Membrane Potential (ΔΨm)

Fluorescent probe JC-1 is a cationic lipophilic dye that can freely pass through the cell membrane and maintains a dynamic balance at both sides of the membrane by changing the cell membrane potential. JC-1 differentially labels mitochondria with high and low Δ*ψ*m by forming J-aggregates or monomers that emit orange-red or green light, respectively. Thus, the fluorescent intensity ratio (A/M) of J-aggregates/J-monomers in mitochondria can be detected to determine early apoptosis.

**Figure 9:**
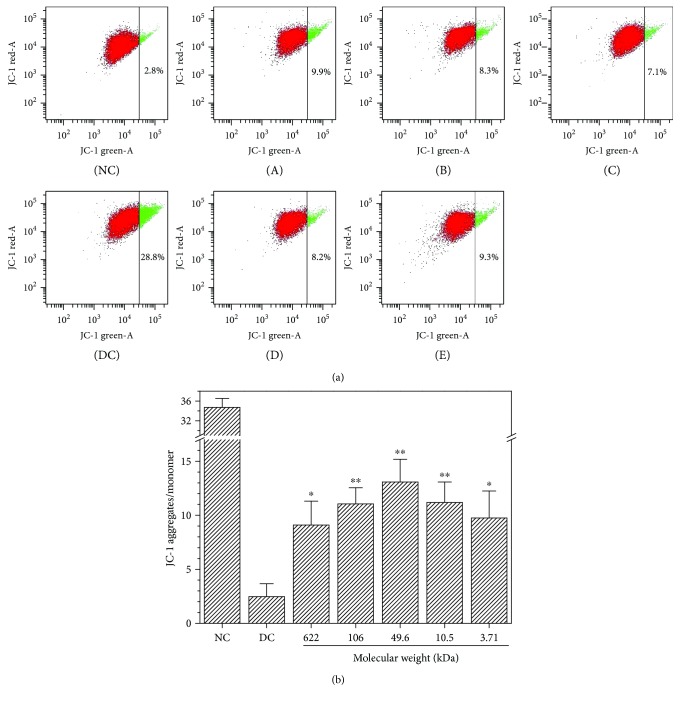
Changes in mitochondrial membrane potential (ΔΨm) of the damaged HK-2 cells after being repaired by GLP with different molecular weights. (a) Dot plot of ΔΨm; (b) changes in the fluorescent intensity ratio (A/M) of J-aggregates/J-monomers in the mitochondria of each repair group. NC: normal control; DC: damaged control by 2.8 mmol/L oxalate; (A) GLP0, (B) GLP1, (C) GLP2, (D) GLP3, and (E) GLP6. GLP concentration: 60 *μ*g/mL. Repaired time: 12 h. Compared to the DC group: ^∗^*p* < 0.05 and ^∗∗^*p* < 0.01.

Figure 9(a) shows the changes in Δ*ψ*m of the damaged HK-2 cells after being repaired by GLP with different molecular weights. The Δ*ψ*m of living cells was high, so the red fluorescence was very strong.  Figure 9(b) shows the changes in the fluorescent intensity ratio of A/M in the mitochondria of each repair group. The A/M ratio in the mitochondria of normal HK-2 cells was higher (34.71), but the A/M ratio decreased to 2.7 in the oxalate-induced damaged group. This finding suggests that Δ*ψ*m evidently decreased in the damaged cells. When the damaged cells were repaired by GLP with different molecular weights, the A/M ratio increased at different degrees (9.1–13.08). The increased Δ*ψ*m caused by GLP2 with a molecular weight of 49.6 kDa was the most obvious, and the A/M ratio increased to 13.08, which is higher than that in the other GLP repair groups.

### 3.11. Changes in Cell Cycle before and after Repair

Propidium iodide (PI) is a DNA double-strand fluorescent dye. The fluorescent intensity produced by the combination of PI and double-strand DNA was positively correlated with DNA content. After intracellular DNA was stained by PI, DNA content can be measured by flow cytometry. Cell cycle can be analyzed according to DNA distribution [29, 30].

**Figure 10:**
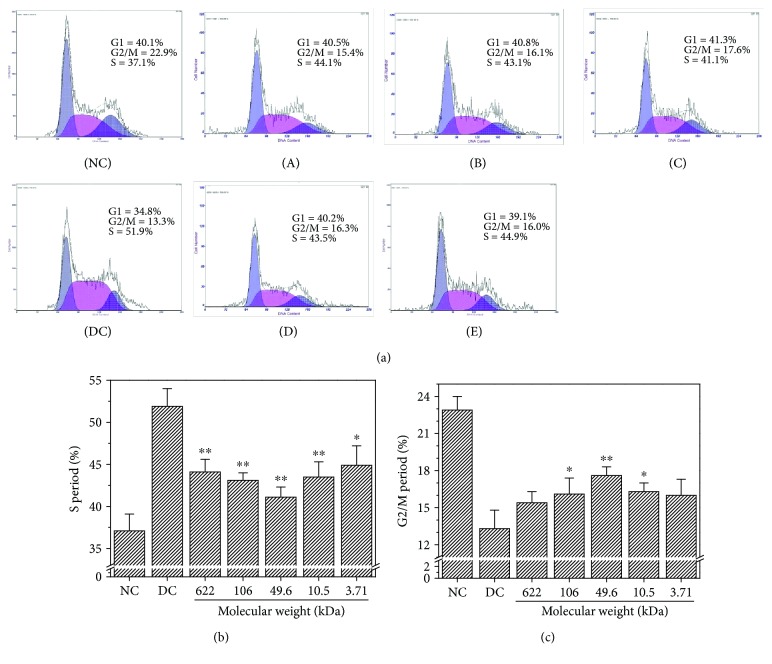
Changes in the cycle of the damaged HK-2 cells after being repaired by GLP fractions with different molecular weights. (a) Cell cycle histogram; (b) the percentage of cells in the S phase; (c) the percentage of cells in the G2/M phase. NC: normal control; DC: damaged control by 2.8 mmol/L oxalate; (A) GLP0, (B) GLP1, (C) GLP2, (D) GLP3, and (E) GLP6. GLP concentration: 60 *μ*g/mL. Repaired time: 12 h. Compared to the DC group: ^∗^*p* < 0.05 and ^∗∗^*p* < 0.01.

Figure 10(a) shows the changes in the cycle of the damaged HK-2 cells after being repaired by GLP fractions with different molecular weights. Compared with the damaged group, the percentage of cells in the S phase evidently decreased (Figure 10(b)), whereas the percentage of cells in the G2/M phase increased (Figure 10(c)). After being repaired by GLP2, the percentage of cells in the S phase was the lowest (41.1%) and those in the G2/M phase increased (17.6%). Hence, GLP2 with molecular weight of 49.6 kDa promoted cells from the S phase to the G2/M phase most efficiently and exhibited the strongest repair effect on the damaged cells.

## 4. Discussion

### 4.1. GLP Degradation and Structure Characterization

Several methods are used for degradation of polysaccharides; such methods include acid hydrolysis, oxidative degradation, and enzymatic methods. The widely used method is H_2_O_2_ degradation, where the dissociation reaction of H_2_O_2_ forms hydroxyl radicals. Hydroxyl radicals are powerful oxidizing substances and can attack the glycosidic bonds of polysaccharides [31]. The degradation reaction of H_2_O_2_ is moderate, and its extent can be controlled without changing the structure of the main chain of polysaccharides. In recent years, H_2_O_2_ degradation has been widely accepted. For example, Hou et al. [32] performed degradation of *Laminaria japonica* fucoidan by changing H_2_O_2_ concentration, reaction temperature, and pH and obtained seven degraded fractions of Mw: 1.0, 3.8, 8.3, 13.2, 35.5, 64.3, and 144.5 kDa. All of these polysaccharide fractions exhibited no significant changes in the major backbone structure and sulfate group content.

**Scheme 1:**
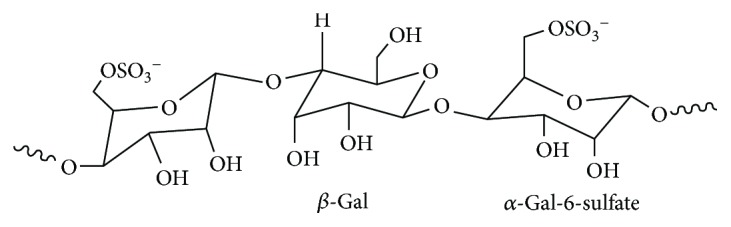
Basic structure unit of GLP.

The structure of polysaccharides is the basis for their biological activity [4, 5, 10, 33]. Galactans from red seaweeds possess structures based on a linear chain of alternating 3-linked *β*-D-galactopyranose residues (A units) and 4-linked *α*-D- or *α*-L-galactopyranose residues or its 3,6-anhydro derivative (B units). These galactans are mainly classified as carrageenans, in which the B unit belongs to the D series, and agarans, in which the B unit is in the L configuration [34]. “Agaran” refers to polysaccharides with a backbone of [→3) *β*-d-Gal-(1→4)-(3,6-An)-*α*-l-Gal]. In the present study, the monosaccharide composition and type of sugar residues of GLP were analyzed by ^1^H NMR, ^13^C NMR, and FT-IR spectroscopy methods. The signals at *δ* 4.43 and 5.34 ppm in the ^1^H NMR spectra correspond to the H1 of *β*-D-galactose and 3,6-*α*-L-galactoptoside, respectively. The signals at *δ* 102.35 and 99.48 ppm in the ^13^C NMR spectra correspond to the anomeric protons of *β*-D-galactose and 3,6-*α*-L-galactoptoside, respectively. The peak at 1370 cm^−1^ in the FT-IR spectra is assigned to ester sulfate, and the peak at 1260 cm^−1^ corresponds to the stretching vibration of S=O [26, 27]. These findings indicate that GLP is a sulfated polysaccharide. Based on the spectroscopy analysis results, GLP mainly consists of *β*-D-galactose and 6-O-sulfate-3,6-L-galactose (Scheme 1). The presence of L-galactose indicates that GLP contains agaran structures. Duarte et al. [35] showed that B units in the L configuration are not of primary importance because of its biological activity, but the substitution of the sulfate group on the agaran backbone affects its biological activity. Thus, the substitution content and position of the sulfate group on the agaran backbone may affect the repair ability of GLP to damaged HK-2 cells.

Polysaccharides contain galactose residue, which is prone to be attacked by free radicals, resulting in fracture of the chain backbone [36]. Thus, GLP can be easily degraded by H_2_O_2_. Depolymerization with H_2_O_2_, a widely used method, changes the side groups of the polysaccharide but does not induce distinct changes in the structures of the main chain (Figure 4) [37]. The FT-IR and ^1^H NMR spectra of GLP before and after degradation were similar (Figure 3, Table 2), and no new peaks appeared. This finding indicated that the degradation of H_2_O_2_ did not significantly affect the overall structure of the polysaccharides. Therefore, the main structure of polysaccharide before and after degradation slightly changed. The molecular weight of the degraded polysaccharides and the H_2_O_2_ concentration showed a negative correlation (Figure 1); this finding provides references for obtaining target polysaccharides with different molecular weights.

As shown in Figures 2 and 3, the contents of the −OSO_3_H and −COOH groups of the polysaccharide fractions slightly increased after degradation because of the following: (1) free radicals produced by H_2_O_2_ during degradation will break the highly compact sugar chain structure of natural polysaccharide, thereby exposing the −OSO_3_H and −COOH groups of the polysaccharide, and (2) the water solubility of the degraded polysaccharide slightly increased and was higher than that of nondegraded GLP0 because of the large molecular weight of the latter, resulting in the concealment of small parts of the acidic groups of polysaccharides [38, 39].

Similar results were reported by previous studies [38, 39]. Zhang et al. [39] obtained polysaccharide fractions with different molecular weights (725, 216, 124, 61.9, and 26.0 kDa) through H_2_O_2_ degradation of the crude *Monostroma latissimum* polysaccharide; the −OSO_3_H group content (21.20%, 22.71%, 24.73%, 25.48%, and 27.28%, resp.) increased with decreasing molecular weight of the polysaccharide. Sun et al. [38] used H_2_O_2_ and Vc to degrade *Pavlova viridis* and *Sarcinochrysis marina* Geitler. When the molecular weight of *P. viridis* decreased from 3645 kDa to 55.0 kDa, the −COOH content increased from 3.41% to 8.78%. The −COOH content increased from 5.82% to 9.99% when the molecular weight of *S. marina* Geitler decreased from 2595 kDa to 169 kDa.

### 4.2. Repair Effect of GLP on Damaged HK-2 Cells

High concentrations of oxalate in urine will cause lipid peroxidation; this phenomenon leads to excessive production of reactive oxygen species (ROS) and MDA (Figure 8(b)) and damage to renal epithelial cells, resulting in enhanced adhesion of urinary crystallites and promoted formation of early microcalculus [40, 41]. Excessive ROS generation resulted in depletion of SOD enzyme activity of cells (Figure 8(a)), indicating that oxalate reduced the antioxidant capacity of cells. GLPs showed significant antioxidant activity and effectively repaired cells against oxidative stress caused by oxalate. The SOD activity was obviously increased when the damaged cells were repaired with 60 *μ*g/mL GLPs, and the MDA content was obviously reduced compared with the injured cells. In addition, the present results indicate that cell viability decreased (Figure 5) and the amount of LDH released into the extracellular matrix increased (Figure 6) when HK-2 cells were damaged by oxalate. After the damaged HK-2 cells were repaired through treatment with various fractions of GLP, the cell viability increased, the amount of LDH released decreased, the cell morphology was improved, and the number of living cells increased (Figure 7). Subha and Varalakshmi [42] reported that sodium pentosan polysulfate can reduce LDH secretion in calculogenic rat urine. Meanwhile, glycosaminoglycan can prevent the changes of cytosolic Ca^2+^ levels in renal tubular epithelial cells induced by oxalic acid and recover the cell morphology [43].

Mitochondrial membrane potential (ΔΨm) is higher in normal cells than that in damaged cells [44]. When the cells were oxidatively damaged by oxalate, the permeability of the mitochondrial membrane increased, which induced Ca^2+^ influx and depolarization of the mitochondria [45], resulting in decreased Δ*ψ*m (Figure 9(b)). GLP fractions with different molecular weights can be used to repair the membrane potential of cells. The increase in Δ*ψ*m is related to the molecular weight of GLP (Figures 8(a)–8(e)); that is, the Δ*ψ*m of the cell was closest to the normal group after being repaired by GLP with molecular weight of 49.6 kDa. Li et al. [46] reported that *Ganoderma atrum* polysaccharide (PSG-1) increased Bcl-2 protein expression in the mitochondria; the polysaccharide inhibited Bax translocation, cytochrome c release, and caspase activation, resulting in an increase in Δ*ψ*m of the cell. *Sparassis crispa* polysaccharide fraction with a molecular weight of 75 kDa reduced the accumulation of reactive oxygen species, blocked Ca^2+^ influx, and prevented depolarization of the mitochondrial membrane potential to protect PC12 cells against L-Glu-induced injury [45]. GLP can repair the mitochondria in damaged cells because −OSO_3_H and −COOH functional groups are rich in GLP, which can scavenge reactive oxygen species [24].

In damaged cells, the percentage of cells in the S phase (51.9%) increased but that in the G2/M phase decreased (Figure 10). The cell cycle was arrested in the S phase. This result could be due to the cell initiated DNA repair when the DNA in the cell was damaged. When DNA cannot repair by itself, the cells cannot enter the G2/M phase and block the S phase [30]. After treatment with GLP with different molecular weights, the percentage of cells in the S phase decreased and that in the G2/M phase increased. Hence, GLP promoted cell cycle progression from the S phase to the G2/M phase and repaired DNA replication. The repair ability on cell cycle progression was associated with the molecular weight of GLP (Figures 9(A)–9(E)), and GLP2 with a molecular weight of 49.6 kDa exhibited the strongest repair ability.

### 4.3. Differences in Repair Effect of Polysaccharides with Different Molecular Weights

The repair effect of GLP on damaged HK-2 cells could be due to polysaccharide molecules, which are dispersed to the damaged cell membrane gap to repair the cell or adhered to the cell membrane to block further oxidative damage by oxalate; the dispersion and adhesion of the polysaccharide are closely correlated with its molecular weight [47].

1. The high molecular weight of polysaccharides limited their physical properties, such as highly compact molecular structure, large molecular size, and low water solubility, resulting in decreased possibility of migrating to the cell membrane [32]; the polysaccharides cannot easily widen the cell membranes to exert their biological effects.
2. For example, the biological availability of low-molecular-weight heparin is higher than that of the ordinary heparin, the former thereby inhibiting atherosclerosis [48]. A previous study on three degraded porphyran fractions (with molecular weights of 29,695, 6893, and 5876 Da) reported that fractions with low molecular weight exhibited strong DPPH radical scavenging ability and antioxidant activity [49]. Sun et al. [50] degraded the crude *Porphyridium cruentum* and obtained six degraded polysaccharide fractions, with molecular weights of 6.53, 256, 606, 802.6, 903.3, and 1002 kDa; the polysaccharide fraction with molecular weight of 6.53 kDa exhibited the optimal effect on enhancing immunomodulatory ability.
3. If the polysaccharide molecular weight is too low, it cannot form an active polymer structure for the biological activity. Liao et al. [6] studied the hypoglycemic effect of GLP with different molecular weights in diabetic mice and discovered that GLP with high molecular weight (such as 121.89 kDa) did not easily pass through the cell membrane to play its biological role; moreover, GLP with low molecular weight (such as 5 kDa) cannot form an active polymer structure for the biological activity, leading to decreased bioactivity. Cai et al. [51] also reported that the anticoagulant activity of GSP-2, with molecular weight of 28 kDa and which was extracted from *G. scabrabunge*, is lower than that of GSP-3, with a high molecular weight of 58 kDa.
4. Polysaccharides with appropriate molecular weight have high freedom degree and small steric hindrance; they require less energy to diffuse into cell membrane breach [47]; therefore, these polysaccharides exhibit great potential to be absorbed by the cell membrane through electrostatic interactions and repair the cell. Thus, degradation of high-molecular-weight polysaccharides into their counterparts with appropriate molecular weight can improve their biological activity [32]. The appropriate molecular weight to obtain optimal bioactivity varies among different plant polysaccharides. For example, Meng et al. [52] reported that polysaccharides with molecular weight of 10–20 kDa exhibited higher hydroxyl radical scavenging activity than did polysaccharide fractions with molecular weight of 43 and 4.7 kDa.

Renal tubular cell injury plays a key role in the pathophysiological processes of renal stone diseases. Abnormally elevated urinary oxalate induces tubular dysfunction or damage, thereby promoting the retention of CaOx crystals. This phenomenon is believed to be a prerequisite for the eventual formation of kidney stones [53]. Our results demonstrated that the GLP fractions can repair oxalate-induced oxidatively damaged HK-2 cells, and their repair ability is correlated with the molecular weight of the fractions. These results may help design structure-based drugs with specific actions on urolithiasis.

## 5. Conclusions

Seven fractions with molecular weights of 106, 49.6, 10.5, 6.14, 5.06, 3.71, and 2.42 kDa were obtained by controlling H_2_O_2_ concentration during degradation of crude GLP. GLP consists of *β*-D-galactose and 6-O-sulfate-3,6-*α*-L-galactose. H_2_O_2_ degradation does not change the structure of GLP polysaccharides, whereas the contents of the −OSO_3_H and −COOH groups of the polysaccharides slightly increased after degradation. Various polysaccharide fractions can repair oxalate-induced oxidatively damaged HK-2 cells. After being repaired by polysaccharides, the cell viability and SOD activity increased, the amount of LDH and MDA released decreased, the cell morphology was gradually restored to normal cells, the mitochondrial membrane potential increased, the percentage of cells in the S phase decreased, and the percentage of cells in the G2/M phase increased. The repair ability of each polysaccharide fraction of GLP is associated with its molecular weight. GLP2 with a molecular weight of 49.6 kDa exhibited a superior repair effect than did other degraded polysaccharide segments and crude polysaccharide. Moreover, polysaccharides with a molecular weight largely deviating from 49.6 kDa elicited weak repair capacity. Our results suggested that degraded GLP fractions, especially GLP2, could be developed into novel anti-stone polysaccharide drugs.

## Figures and Tables

**Table 1:** Degradation conditions and physico-chemical properties of crude *G. lemaneiformis* polysaccharide and seven degraded polysaccharide fractions.

GLP fraction	Concentration of H_2_O_2_/%	Intrinsic viscosity *η* (mL/g)	Mean molecular weight *M*_r_ (kDa)	Yield (%)	–OSO_3_H content (%)	–COOH content (%)
GLP0	0	1039 ± 42	622 ± 35		13.07	1.26
GLP1	0.1	298.8 ± 37.8	106 ± 15	63.5	13.37	1.27
GLP2	0.4	168.3 ± 12.0	49.6 ± 4.8	58.4	13.41	1.28
GLP3	1	54.9 ± 1.4	10.5 ± 3.9	56.0	13.46	1.28
GLP4	3	37.4 ± 1.5	6.14 ± 0.35	52.1	13.55	1.36
GLP5	6	33.7 ± 0.9	5.06 ± 0.2	61.3	13.55	1.54
GLP6	10	26.0 ± 0.9	3.71 ± 0.18	53.0	13.56	1.76
GLP7	15	19.7 ± 0.9	2.42 ± 0.16	60.0	13.46	1.77

**Table 2:** FT-IR characteristic absorption peaks of GLP.

Sample	Molecular weight *M*_r_ (kDa)	Content of −OSO_3_H (%)	Content of −COOH (%)	Characteristic absorption peak (cm^−1^)			
				−OH	−COOH	−OSO_3_	Sugar ring
GLP0	622 ± 35	13.07	1.26	3432	1643	1380, 1257	2928, 1048
GLP2	49.6 ± 4.8	13.41	1.28	3421	1604	1380, 1263	2928, 1053
GLP3	10.5 ± 3.9	13.46	1.30	3408	1604	1380, 1258	2920, 1043
GLP7	2.42 ± 0.16	13.56	1.77	3409	1609	1380, 1268	2920, 1043

**Table 3:** ^13^C NMR chemical shift data of GLP.

Monosaccharide types	^13^C chemical shift (ppm)					
	C-1	C-2	C-3	C-4	C-5	C-6
*β*-D-Galactose	102.28	71.14	78.28	71.46	73.96	59.73
6-O-Sulfate-L-*α*-galactopyranose	99.50	72.82	74.74	72.76	73.26	60.35
